# Relationship Between Diet Quality and Antihypertensive Medication Intensity Among Adults With Metabolic Syndrome-Associated High Blood Pressure

**DOI:** 10.1016/j.cjco.2023.09.016

**Published:** 2023-09-28

**Authors:** Lise Leblay, Amélie Bélanger, Clémence Desjardins, Mathieu Filiatrault, Jean-Sébastien Paquette, Jean-Philippe Drouin-Chartier

**Affiliations:** a Centre Nutrition, Santé et Société (NUTRISS), Institut sur la Nutrition et les Aliments Fonctionnels (INAF), Université Laval, Québec City, Québec, Canada; b Faculté de Pharmacie, Université Laval, Québec City, Québec, Canada; c Département de médecine familiale et de médecine d’urgence, Faculté de Médecine, Université Laval, Québec City, Québec, Canada; d VITAM, Centre de recherche en santé durable, Université Laval, Québec City, Québec, Canada; e Centre Hospitalier Régionale de Lanaudière, Saint-Charles-Borromée, Québec City, Québec, Canada

## Abstract

**Background:**

Management of high blood pressure (BP), a key feature of the metabolic syndrome (MetS), relies on diet and medication. Whether these modalities are used as complements has never been evaluated in real-world settings. This study assessed the relationship between diet quality and antihypertensive medication intensity among adults with MetS-associated high BP.

**Methods:**

This cross-sectional study included 915 adults with MetS-associated high BP from the CARTaGENE cohort (Québec, Canada), of whom 677 reported using BP-lowering medication. Antihypertensive medication intensity was graded per the number of BP-lowering classes used simultaneously. Diet quality was assessed using the **D**ietary **A**pproach to **S**top **H**ypertension (DASH) score.

**Results:**

No evidence of a relationship between antihypertensive medication intensity and diet quality was found (β for each additional antihypertensive = –0.05; 95% CI, –0.35; 0.26 DASH score points). However, among men aged < 50 years and women aged < 60 years, the DASH score was inversely associated with medication intensity (β = –0.72; 95% CI, –1.24, –0.19), whereas this relationship tended to be positive among older participants (β = 0.32; 95% CI, –0.05, 0.69). Among participants with low Framingham risk score, the DASH score was inversely associated with medication intensity (β = –0.70; 95% CI, –1.31, –0.09), but no evidence of an association was found among individuals at moderate (β = 0.00; 95% CI, –0.45, 0.45) or high (β = 0.30, 95% CI, –0.24, 0.84) risk.

**Conclusions:**

In this cohort of adults with MetS-associated high BP, there was an overall lack of complementarity between diet quality and BP-lowering medication, especially among younger individuals and those with a lower risk for cardiovascular disease for whom diet quality was inversely associated with intensity of medication.

## Résumé

**Introduction:**

La prise en charge de la pression artérielle (PA) élevée, l’une des caractéristiques principales du syndrome métabolique (SMet), repose sur une saine alimentation et l'usage de médicaments hypotenseurs. Il n’a jamais été évalué si ces modalités étaient utilisées de façon complémentaire en contexte réel. La présente étude portait sur la relation entre la qualité de l'alimentation et la médication antihypertensive au sein d'adultes ayant une PA élevée associée au SMet.

**Méthodes:**

Cette étude transversale regroupait 915 adultes de la cohorte CARTaGENE (Québec, Canada) ayant une PA élevée associée au SMet, dont 677 ont rapporté prendre des médicaments abaissant la PA. L’intensité de la médication antihypertensive a été évaluée par le nombre de classes de médicaments abaissant la PA utilisées simultanément. La qualité du régime alimentaire a été évaluée à l’aide du score **D**ietary **A**pproache to **S**top **H**ypertension (DASH).

**Résultats:**

Aucune évidence d’une relation entre l’intensité des médicaments antihypertenseurs et la qualité de l'alimentation n’a été observée (β pour chaque antihypertenseur supplémentaire = –0,05 ; intervalle de confiance [IC] à 95 %, –0,35 ; 0,26 point au score DASH). Toutefois, chez les hommes âgés < 50 ans et chez les femmes âgées < 60 ans, le score DASH était inversement associé à l’intensité des médicaments (β = –0,72 ; IC à 95 %, –1,24, –0,19), alors que cette relation avait tendance à être positive chez les participants plus âgés (β = 0,32 ; IC à 95 %, –0,05, 0,69). Chez les participants qui avaient un score de risque de Framingham faible, le score DASH était inversement associé à l’intensité des médicaments (β = –0,70 ; IC à 95 %, –1,31, –0,09), mais aucune évidence d’une association n’a été observée chez les individus qui avaient un risque modéré (β = 0,00 ; IC à 95 %, –0,45, 0,45) ou élevé (β = 0,30, IC à 95 %, –0,24, 0,84).

**Conclusions:**

Au sein de cette cohorte d’adultes qui avaient une PA élevée associée au SMet, aucune évidence de complémentarité entre la qualité de l'alimentation et l'intensité de la médication antihypertensive n'a été observée, particulièrement chez les individus plus jeunes et chez ceux exposés à un risque plus faible de maladies cardiovasculaires pour qui la qualité du régime alimentaire était inversement associée à l’intensité des médicaments.

Metabolic syndrome (MetS) refers to the cluster of metabolic abnormalities associated with abdominal obesity.^1^ These include dysregulated glucose homeostasis, an atherogenic dyslipidemia, and hemodynamic changes increasing blood pressure (BP).^2^ MetS affects about 1 in 3 adults in North America and drastically increases the risk of cardiovascular diseases (CVDs).^2^^,^^3^ High BP has repeatedly been reported as the most prevalent feature of MetS.^4^ Notably, individuals with MetS and concomitant high BP exhibit higher systolic and diastolic BP as well as higher risk of CVD than individuals with high BP but without MetS.^4^, ^5^, ^6^ These data underline the crucial importance of adequately controlling BP in MetS.

The cornerstone of management of high BP relies on the complementarity of lifestyle modification, for which diet is a key component, and medication use.^7^ On the one hand, the **D**ietary **A**pproach to **S**top **H**ypertension (DASH) trial has demonstrated the hypotensive effects of a diet rich in vegetables, fruits, low-fat dairy products, whole grains, fish, legumes, and nuts.^8^ Subsequent studies confirmed that such dietary pattern is not only associated with lower BP but also with lower incidence of CVD.^9^ On the other hand, when lifestyle management is not sufficient to normalize BP, BP-lowering medication is recommended. Several BP-lowering agents are available. As such, if monotherapy induces unsufficient decreases in BP, the intensity of the pharmacologic therapy can be increased by combining different classes.^7^ Still, in spite of the numerous dietary and pharmacologic options to control BP, national rates of adequate hypertension control have declined in the past decade in both Canada and the United States.^10^^,^^11^ These statistics raise questions on the complementarity between nutritional and pharmacologic management of high BP, especially as the initiation of BP-lowering medication has been associated with unfavourable lifestyle changes.^12^ Indeed, prospective data from Finland showed that individuals who initiated BP-lowering medications were more likely to decrease their practice of physical activity subsequently compared with noninitiators.^12^ The underlying reason would be related—at least in part—to the perceived effectiveness of medication, which would represent a barrier to favourable lifestyle modifications.^12^ Ultimately, substituting medication for lifestyle management is likely to alter control of BP and lead to intensification of drug treatment, which may increase the risk of adverse effects and nonadherence, further fuelling this cycle.^13^ Still, to our knowledge, no study has assessed the relationship between intensity of BP-lowering medication and diet quality among adults with MetS-associated high BP.

The objective of this study was to assess the relationship between diet and BP-lowering medication use in the management of MetS-associated high BP among adults from the Province of Québec (Canada). Specifically, we first evaluated the relationship between diet quality, using the DASH score, and BP-lowering pharmacotherapy intensity. Second, we assessed how these 2 modalities were associated with control of BP.

## Methods

The protocol was reviewed and approved by Laval University Ethics Committee and CARTaGENE Sample and Data Access Committee.

### Study population

This study is a cross-sectional analysis within the CARTaGENE Québec population-based cohort (Canada).^14^ At study inception, 43,037 Québec residents aged between 40 to 69 years were recruited to participate in CARTaGENE. Recruitment took place during 2 phases (A: n = 19,068, 2009-2010; B: n = 23,969, 2013-2014). Participants were randomly selected from provincial health insurance registries to be representative of the 2006 Québec population based on age, sex, and area of residence. CARTaGENE adhered to the Declaration of Helsinki, and all participants signed informed consent forms at the time of inclusion in the study.^14^

The current analysis leverages data from phase A, as diet was only assessed among participants of this phase. For phase A, data collection was conducted during in-person interviews in 2009 and 2010. The interview included identification; consent; self-administered sociodemographic and lifestyle questionnaire; interviewer-administered health questionnaire; physical measurements; and biospecimen collection.^14^, ^15^, ^16^ In 2012, phase A participants were invited by mail to complete a food frequency questionnaire (FFQ) from home, which ended being returned by ∼10,000 individuals.^14^

For the current study, inclusion criteria were having MetS (per the harmonized definition)^17^; having self-reported a history of high BP or a hypertension diagnosis (ie, all included participants were aware that they had high BP); having adequately completed the FFQ (ie, < 40% of blank items); having reported plausible energy intakes in the FFQ (ie, women: 500-3500 kcal per day; men: 800-4200 kcal per day); and having had BP measured during the in-person interview. Individuals having histories of diabetes, CVD, or cancer or who were pregnant at the moment of data collection were excluded. A total of 915 adults were included in the study (Supplemental Fig. S1).

### Assessment of diet and diet quality

Diet was assessed using the Canadian Dietary History Questionnaire II (C-DHQ II).^18^, ^19^, ^20^, ^21^, ^22^ This FFQ was initially developed and validated by the US National Cancer Institute. This FFQ assesses dietary intakes over the 12 months preceding its completion, addressing the frequency of consumption of 153 foods and the portion size usually consumed.

Diet quality was assessed using the DASH score.^23^ This score is calculated based on intake of the 8 key food groups and nutrients in the DASH diet: whole fruits; whole vegetables; nuts and legumes; low-fat dairy products; whole grains; red and processed meats; sugar-sweetened beverages (SSBs); and sodium. For each component, participants were classified into quintiles according to their daily intake. For whole fruits, whole vegetables, nuts and legumes, low-fat dairy products, and whole grains, the score attributed to each participant was equivalent to its quintile ranking. For red and processed meats, SSBs, and sodium, the lowest quintile of intake was attributed a score of 5 points, whereas the highest quintile was given 1 point. Scores were summed to obtain the DASH score, which could range from 8 (minimum adherence) to 40 (maximum adherence).^23^

### Assessment of blood pressure-lowering medication use and intensity

Information on BP-lowering medication use was obtained from the health questionnaire in which participants had to self-report the types and the dosages of the medication they used. A previous study within CARTaGENE demonstrated the high agreement between self-reported medication and claim prescription records (kappa [κ] > 0.80).^24^ We graded the intensity of BP-lowering medication according to the number of BP-lowering medication classes used simultaneously, in accordance with the International Society of Hypertension guidelines^7^ and as done in previous studies.^25^^,^^26^ In sensitivity analyses, we graded intensity of medication using the number of antihypertensive pills to take into account polypharmacy burden as well as the fact that some classes can be combined in a single pill.^27^ Finally, we assessed medication intensity using the Therapeutic Intensity Score (TIS).^28^ This score takes into account the number and the types of medication, the prescribed daily dose, and maximum recommended daily dose. A higher TIS indicates greater intensity of medication. The TIS was calculated among the subgroup of participants who provided complete information about the dosages of their BP-lowering therapy (n = 594; ie, 238 individuals not using medication [TIS = 0], and 356 individuals using medication [TIS > 0]).

### Assessment of blood pressure and other physical and biochemical parameters

Systolic and diastolic BP were measured during the in-person interview, after 10 minutes of seated rest in an isolated room, with previous upper-arm measurement to determine adequate BP cuff size. Data were reported as the average of 3 measurements performed at 2-minute intervals using an automated oscillometric device (Colin Press-Mate Prodigy II Vital Signs Monitor OM-2200, Orlando, Florida, USA).^14^

Concentrations of total-cholesterol, HDL-cholesterol, triglycerides, and glucose were measured from the fasting plasma sample collected during the in-person interview.^14^ Quality assurance tests during the optimization phase demonstrated that all parameters were measured with test-retest reliability > 90%.^14^ Waist circumference was measured twice (SECA 200 measuring tape [SECA Precision for Health, Hamburg, Germany]). Participants’ height was also measured twice (SECA 214 portable stadiometer). A digital scale was used to measure the weight of each participant.

### Statistical analyses

Statistical analyses were performed using SAS software, version 9.4 (SAS Institute, Cary, North Carolina, USA). All statistical tests were 2-sided with a significance threshold set at *P* < 0.05. First, we assessed the relationship between diet quality (ie, DASH score) or dietary intakes (ie, subscores and daily intakes of DASH components) and the intensity of antihypertensive drug therapy (ie, the number of classes used simultaneously) using linear regression models (general linear model [GLM] procedure). Models were adjusted for gender (men, women), age (years), annual household income (< $10,000; $10,000-$24,999; $25,000-$49,999; $50,000-$74,999; $75,000-$99,999; $100,000-$149,999; $150,000-$199,999; > $200,000); body mass index [BMI] kg/m^2^); smoking status (never/ever/current), physical activity level (low/moderate/high), self-reported history of high blood cholesterol (no/yes), alcohol consumption (grams per day), and energy intake (kcal per day). A post hoc F test was used to compute achieved power of this analysis using GPower software, version 3.1 (GPower ApS, Hinnerup, Denmark). In sensitivity analyses, these analyses were first repeated by comparing participants according to whether or not they were using BP-lowering medication. We also repeated the main analyses by modelling intensity of BP-lowering medication continuously as the number of BP-lowering pills used simultaneously and by using the TIS. The latter narrowed down the sample to participants among whom the TIS could be calculated (n = 594).

Next, we conducted prespecified stratified analyses to assess whether the relationship between the DASH score and intensity of antihypertensive medication differed according to gender (women vs men), age (men < 50 and women < 60 years vs men ≥ 50 and women ≥ 60 years), education level (high school or less vs college or university), annual household income (< $50,000 vs ≥ $50,000), self-reported history of high blood cholesterol (no vs yes), smoking status (never vs past vs current), or Framingham risk score (low vs moderate vs high). Evidence of differential associations was assessed using the *P* value of the interaction term between medication intensity (ie, number of BP-lowering classes) and the stratification variable. The sex/gender-specific approach in the age stratification reflects the thresholds associated with higher age-related risk of CVD.^7^^,^^29^

The final set of analyses focused on control of BP. We assessed the relationships between diet, intensity of BP-lowering medication, and systolic and diastolic BP using linear regression models (GLM procedure). Systolic and diastolic BP was included as dependent variables in separate models. The covariable structure was the same as described here. Models with diet quality (DASH score) as the main exposure were adjusted for intensity of BP-lowering medication, and vice-versa. In analyses with specific components of the DASH score as the main exposures, we mutually adjusted for each food group. In analyses with antihypertensive medication intensity as the main exposure, Tukey-Kramer’s multiple comparison tests were used to identify between-group statistical differences.

For all statistical models, the normality of the linear models was assessed using the distribution of the scaled residual values. For cases in which these were not normally distributed, we used the Box-Cox approach (transformation regression [TRANSREG] procedure) to identify the transformation that allowed normalization of the model scaled residual values.

## Results

Characteristics of the 915 participants with MetS-associated high BP included in the study (women, n = 463; men, n = 452) according to the intensity of antihypertensive medication are presented in Table 1. Approximately one-fourth of included participants were not using antihypertensive medication. Most participants were using either 1 or 2 different BP-lowering drugs. Individuals using ≥ 3 types of antihypertensive medications tended to be older, were more likely to have an annual household incomes of < $49,999, and to be past smokers. They also had a higher waist circumferences and BMIs. Angiotensin II receptor blockers were mostly used among individuals treated with monotherapy. Diuretics and calcium channel blockers were mostly used among individuals using 2 or ≥ 3 different BP-lowering medications, respectively. The mean TIS increased along with the number of medication classes used simultaneously. Comparing participants according to the number of BP-lowering pills they used yielded similar observations (Supplemental Table S1).

**Table 1:** Characteristics of the 915 participants with MetS-associated high blood pressure according to the intensity of antihypertensive medication therapy∗

Characteristics	Number of antihypertensive medication classes used simultaneously			
	0	1	2	≥ 3†
Participants, n (%)	238 (26.0)	385 (42.1)	217 (23.7)	75 (8.2)
Age, years	56.7 ± 7.4	57.8 ± 7.4	58.5 ± 7.1	59.0 ± 6.9
Sex/gender, n (%)				
Men	123 (51.7)	188 (48.8)	103 (47.5)	38 (50.7)
Women	115 (48.3)	197 (51.2)	114 (52.5)	37 (49.3)
Annual household income, n (%)				
Less than $49,999	86 (36.1)	126 (32.7)	82 (37.8)	36 (48.0)
$50,000-$99,999	88 (37.0)	182 (47.3)	99 (45.6)	30 (40.0)
More than $100,000	64 (26.9)	77 (20.0)	36 (16.6)	9 (12.0)
Self-reported history of BP cholesterol, n (%)	85 (35.7)	150 (39.0)	101 (46.5)	25 (33.3)
Smoking status, n (%)				
Never	86 (36.1)	175 (45.5)	90 (41.5)	27 (36.0)
Past	115 (48.3)	162 (42.1)	102 (47.0)	40 (53.3)
Current	37 (15.6)	48 (12.5)	25 (11.5)	8 (10.7)
Alcohol consumption, grams/day	12.2 ± 18.8	11.4 ± 20.0	10.9 ± 14.4	11.7 ± 16.9
Energy intake, kcal/day	1,966 ± 701	1,957 ± 721	1,865 ± 694	1,892 ± 663
DASH score, points	23.9 ± 4.8	24.1 ± 4.4	24.1 ± 4.5	23.5 ± 5.4
Physical activity level, n (%)				
Low	40 (16.8)	65 (16.9)	47 (21.7)	16 (21.3)
Moderate	111 (46.6)	179 (46.5)	102 (47.0)	26 (34.7)
High	87 (36.6)	141 (36.6)	68 (31.3)	33 (44.0)
Waist circumference, cm	101 ± 14	101 ± 13	103 ± 12	106 ± 14
Body mass index, kg/m^2^	30.0 ± 5.4	30.0 ± 5.2	31.3 ± 5.6	33.1 ± 7.4
Systolic BP, mm Hg	137 ± 17	132 ± 14	130 ± 16	130 ± 16
Diastolic BP, mm Hg	82 ± 11	78 ± 10	77 ± 10	75 ± 10
Framingham risk score				
Low (< 10%)	53 (22.3)	102 (26.5)	62 (28.6)	19 (25.3)
Moderate (10-19%)	97 (40.8)	158 (41.0)	82 (37.8)	37 (49.3)
High (≥ 20%)	88 (37.0)	125 (32.5)	73 (33.6)	19 (25.3)
BP-lowering medication class, n (%)				
Alpha adrenergic receptors‡	0 (0)	5 (1.3)	3 (1.4)	5 (6.7)
Angiotensin-converting enzyme inhibitors	0 (0)	71 (18.4)	38 (17.5)	16 (21.3)
Angiotensin II receptor blockers	0 (0)	153 (39.7)	53 (24.4)	21 (28.0)
Beta-adrenergic receptor blockers	0 (0)	54 (14.0)	55 (25.4)	35 (46.7)
Calcium channel blockers	0 (0)	53 (13.8)	46 (21.2)	58 (77.3)
Diuretics	0 (0)	49 (12.7)	65 (30.0)	27 (36.0)
Number of BP-lowering pills, n (%)				
1	0 (0)	385 (100)	87 (40.1)	0 (0)
2	0 (0)	0 (0)	129 (59.5)	34 (45.3)
3 or more	0 (0)	0 (0)	1 (0.5)	41 (54.7)
Therapeutic intensity score§	0	0.48 ± 0.34	0.76 ± 0.50	0.80 ± 0.44

BP, blood pressure; DASH, Dietary Approaches to Stop Hypertension; MetS, metabolic syndrome; SD, standard deviation.

∗ Continuous variables are presented as mean ± SD. Categorical variables are presented as count (percent).

† Three classes, n = 59; 4 classes, n = 12; 5 classes, n = 3; 6 classes, n = 1.

‡ A total of 9 of 13 individuals using alpha-adrenergic receptors were women.

§ Data on the Therapeutic Intensity Score were available for n = 594 of 915 participants (no medication: n = 238; 1 class: n = 204; 2 classes: n = 113; ≥ 3 classes: n = 39).

**Figure 1:**
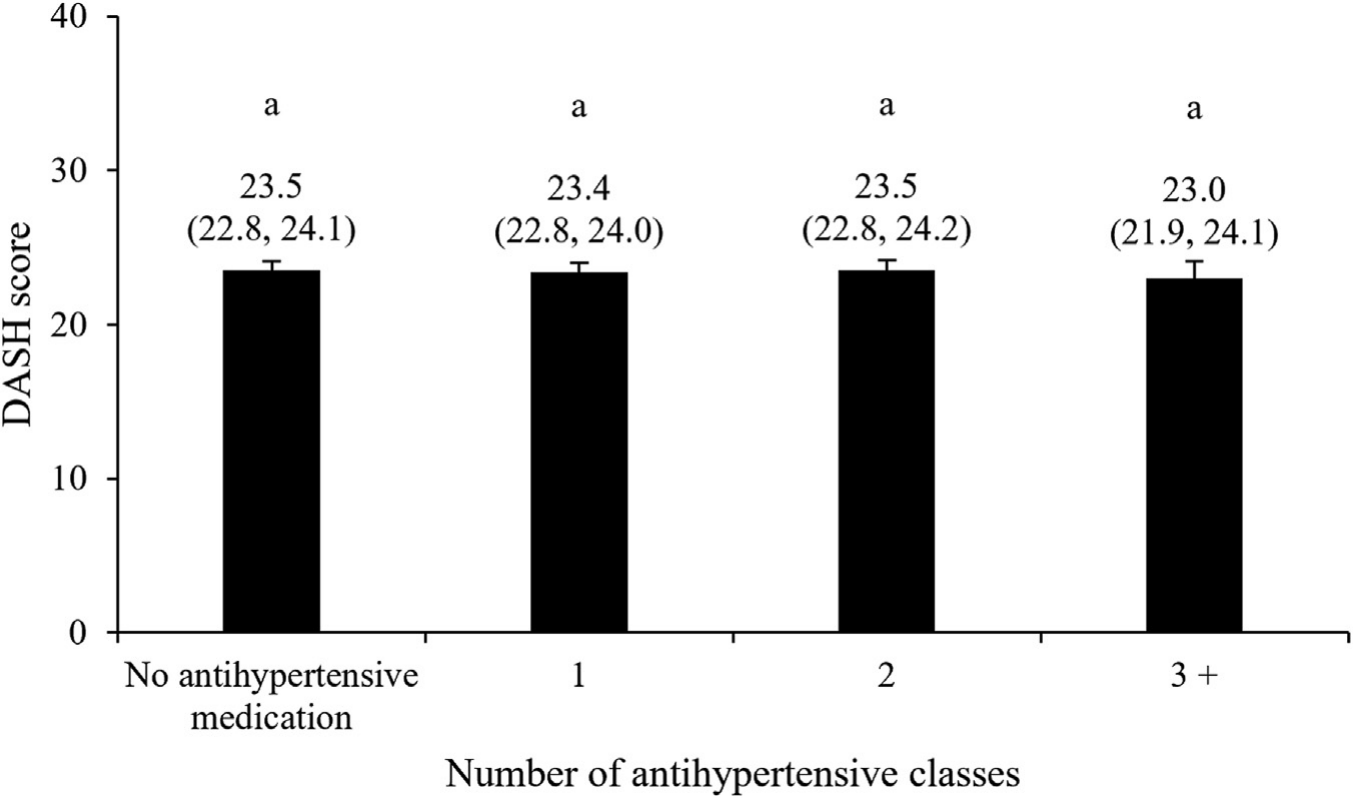
Dietary Approaches to Stop Hypertension (DASH) score according to antihypertensive medication intensity. Data are presented as mean (95% confidence interval), following adjustments for gender (men, women), age (years), annual household income (< $10,000; $10,000-$24,999; $25,000-$49,999; $50,000-$74,999; $75,000-$99,999; $100,000-$149,999; $150,000-$199,999; > $200,000), body mass index (kg/m^2^), smoking status (never/ever/current), physical activity level (low/moderate/high), self-reported history of high blood cholesterol (yes/no), alcohol consumption (grams per day), and energy intake (kcal per day). The number of antihypertensive medication class was treated as a categorical variable in this model (0; 1; 2; 3+). *P* value for between-group differences associated with the number of blood pressure-lowering medication classes = 0.86. Groups with different superscript letters are statistically different (Tukey-Kramer’s multiple comparison test, *P* < 0.05).

We found no evidence of a difference in the DASH score according to the intensity of BP-lowering medication, assessed using the number of antihypertensive medication classes used simultaneously (Fig. 1, *P* = 0.86; Table 2, *P* = 0.76). The lack of evidence of a relationship between the DASH score and medication intensity was not caused by low statistical power, as the achieved statistical power of this model exceeded 0.99. The same observation was made when the intensity of antihypertensive medication was assessed using the number of BP-lowering pills (Supplemental Table S2**)** or the TIS (Supplemental Table S3). We also observed no evidence of a difference in the DASH score when we compared individuals using antihypertensive medication with those not using medication (Supplemental Table S4). However, evidence of associations between the DASH score and intensity of BP-lowering medication was found when participants were stratified according to age (*P* interaction = 0.002) or Framingham risk score (*P* interaction = 0.05) (Table 3). Among participants of younger age (ie, men < 50 years; women < 60 years), the DASH score was inversely associated with medication intensity (*P* = 0.007), although a statistical trend suggested that it was positively associated with the number of antihypertensive classes used simultaneously among older participants (*P* = 0.09). Among participants with low Framingham risk scores, the DASH score was inversely associated with the number of antihypertensive classes used simultaneously (*P* = 0.02), whereas no evidence of association was found among individuals at moderate (*P* = 0.99) or high (*P* = 0.28) risk for CVD.

**Table 2:** Differences in the DASH score and related dietary components associated with each additional BP-lowering drug class among the 915 included participants with MetS-associated high BP∗

Dietary components	β coefficient (95% CI) associated with each additional BP-lowering drug class	*P* value
DASH total score	–0.05 (–0.35, 0.26)	0.76
Whole grains		
DASH subscore	–0.01 (–0.10, 0.09)	0.87
Servings per day	–0.01 (–0.07, 0.05)	0.98
Whole vegetables		
DASH subscore	–0.03 (–0.11, 0.06)	0.49
Servings per day	–0.01 (–0.23, 0.20)	0.54
Whole fruits		
DASH subscore	–0.05 (–0.14, 0.04)	0.27
Servings per day	–0.02 (–0.11, 0.07)	0.30
Low-fat dairy products		
DASH subscore	0.10 (0.00, 0.19)	0.04
Servings per day	0.02 (–0.07, 0.11)	0.10
Red and processed meats		
DASH subscore	0.02 (–0.06, 0.10)	0.60
Servings per day	0.01 (–0.03, 0.05)	0.85
Nuts and legumes		
DASH subscore	–0.07 (–0.16, 0.02)	0.15
Servings per day	–0.04 (–0.08, 0.01)	0.11
Sugar-sweetened beverages		
DASH subscore	–0.01 (–0.10, 0.09)	0.80
Servings per day	0.02 (–0.19, 0.23)	0.94
Sodium		
DASH subscore	–0.01 (–0.06, 0.05)	0.55
mg per day	31.2 (–10.7, 73.1)	0.33

BMI, body mass index; BP, blood pressure; CI, confidence interval; DASH, Dietary Approaches to Stop Hypertension; MetS, metabolic syndrome.

∗ Data are presented as β coefficient (95% confidence interval), in DASH points or daily intakes, associated with each additional BP-lowering medication class (continuous variable). Models were adjusted for gender (men, women), age (years), annual household income (< $10,000; $10,000-$24,999; $25,000-$49,999; $50,000-$74,999; $75,000-$99,999; $100,000-$149,999; $150,000-$199,999; > $200,000), BMI (kg/m^2^), smoking status (never, ever, current), alcohol consumption (grams per day), energy intake (kcal per day), physical activity level (low/moderate/high) and self-reported history of high blood cholesterol (yes/no).

**Table 3:** Differences in the DASH score associated with each additional BP-lowering medication class among the 915 included participants with MetS-associated high BP, after stratification by key characteristics∗

Stratification	β coefficient (95% CI) associated with each additional BP-lowering medication class	*P* value	*P* value for interaction
Gender			
Women	–0.24 (–0.68, 0.20)	0.29	0.24
Men	0.12 (–0.29, 0.53)	0.57	
Age			
Men: < 50 years Women: < 60 years	–0.72 (–1.24, –0.19)	0.007	0.001
Men: ≥ 50 years Women: ≥ 60 years	0.32 (–0.05, 0.69)	0.09	
Education level			
High school or less	0.07 (–0.48, 0.63)	0.79	0.61
College or university	–0.10 (–0.45, 0.26)	0.60	
Annual household income			
< $50,000	–0.17 (–0.65, 0.30)	0.47	0.52
≥ $50,000	0.03 (–0.36, 0.42)	0.89	
Body mass index			
< 30 kg/m^2^	–0.13 (–0.57, 0.32)	0.58	0.55
≥ 30 kg/m^2^	0.06 (–0.35, 0.47)	0.78	
Smoking status			
Never	–0.08 (–0.59, 0.43)	0.77	0.98
Past	–0.04 (–0.45, 0.37)	0.85	
Current	0.02 (–0.86, 0.89)	0.97	
Self-reported history of BP cholesterol			
No	0.09 (–0.29, 0.47)	0.63	0.23
Yes	–0.28 (–0.78, 0.21)	0.26	
Framingham risk score			
Low (< 10%)	–0.70 (–1.31, –0.09)	0.02	0.05
Moderate (10%-19%)	0.00 (–0.45, 0.45)	0.99	
High (≥ 20%)	0.30 (–0.24, 0.84)	0.28	

BMI, body mass index; BP, blood pressure; CI, confidence interval; DASH, Dietary Approaches to Stop Hypertension; MetS, metabolic syndrome.

∗ Data are presented as β coefficient (95% confidence interval), in DASH points, associated with each additional BP-lowering medication class (continuous variable). Models were adjusted for sex/gender (men, women), age (years), annual household income (< $10,000; $10,000-$24,999; $25,000-$49,999; $50,000-$74,999; $75,000-$99,999; $100,000-$149,999; $150,000-$199,999; > $200,000), BMI (kg/m^2^), smoking status (never/ever/current), alcohol consumption (grams per day), energy intake (kcal per day), physical activity level (low/moderate/high), and self-reported history of high blood cholesterol (yes/no).

With regard to the dietary components of the DASH score, we found no evidence of associations between the consumption of these foods or nutrients, assessed using either DASH subscores or daily intakes (servings), and the number of BP-lowering medications used concomitantly (Table 2). The only exception was related to low-fat dairy product intakes, for which the DASH subscore was positively associated with the number of antihypertensive classes used simultaneously (*P* = 0.04). Observations were similar when intakes of the DASH score components were assessed in relation with the number of BP-lowering pills (Supplemental Table S2). When we repeated these analyses using the TIS, the relationship with low-fat dairy intake was no longer observed, but whole vegetable consumption was found to be inversely associated with TIS (Supplemental Table S3). When we compared intakes of participants according to whether or not they used antihypertensive medication (Supplemental Table S4), individuals using antihypertensive medication consumed more low-fat dairy products (*P* = 0.04) and fewer whole vegetables (*P* = 0.03) than those not using medication.

**Figure 2:**
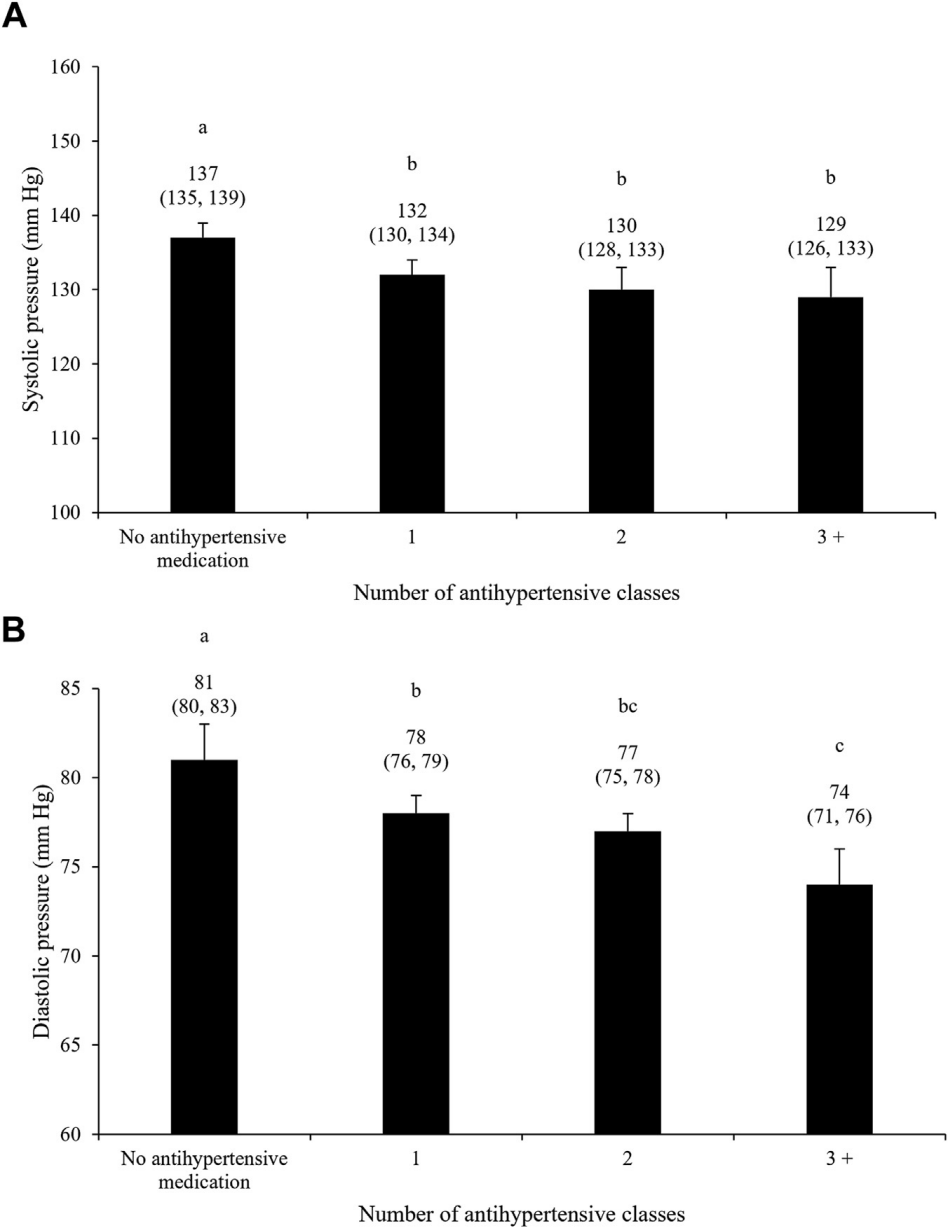
Systolic (**A**) and diastolic (**B**) blood pressure according to antihypertensive medication intensity. Data are presented as mean (95% confidence interval). Models were adjusted for sex (male, female), age (years), annual household income (< $10,000; $10,000-$24,999; $25,000-$49,999; $50,000-$74,999; $75,000-$99,999; $100,000-$149,999; $150,000-$199,999; > $200,000), body mass index (kg/m^2^), smoking status (never/ever/current), alcohol consumption (grams per day), energy intake (kcal per day), physical activity level (low/moderate/high), self-reported history of high blood cholesterol (no/yes), and DASH score. The number of antihypertensive medication classes was treated as a categorical variable in this model (0; 1; 2; 3+). Groups with different superscript letters are statistically different (Tukey-Kramer’s multiple comparison test, *P* < 0.05). DASH, Dietary Approaches to Stop Hypertension.

With regard to control of BP, a statistical trend suggested an inverse relationship between the DASH score and diastolic BP (*P* = 0.10), but no evidence of a relationship was observed with systolic BP (*P* = 0.20; Table 4). Intakes of whole grains, in servings per day, were inversely associated with both systolic and diastolic BP (*P* = 0.01 for both), whereas DASH subscore associated with SSB intakes tended to be inversely associated with both systolic and diastolic BP (*P* = 0.06 for both). Participants using antihypertensive medication had significantly lower systolic and diastolic BP compared with individuals not using medication (*P* < 0.05, for all), but only differences in diastolic BP, not in systolic BP, were found among individuals using 1, 2, or ≥ 3 BP-lowering medications simultaneously (Fig. 2). Finally, the TIS was inversely associated with both systolic and diastolic BP (β systolic BP = –5.13, 95% confidence interval [CI], –8.00, –2.26 mm Hg; β diastolic BP = –3.27, 95% CI, –5.21, –1.33 mm Hg).

**Table 4:** Relationships among the DASH score, the DASH score components, and systolic and diastolic BP among the 915 participants with MetS-associated high BP included in the study∗

Dietary components	Systolic BP (mm Hg)	*P* value	Diastolic BP (mm Hg)	*P* value
Total DASH score	–0.14 (–0.36, 0.09)	0.20	–0.12 (–0.27, 0.03)	0.10
Whole grains				
DASH subscore	–0.28 (–1.04, 0.49)	0.39	–0.36 (–0.87, 0.16)	0.15
Servings per day	–1.44 (–2.59, –0.30)	0.01	–0.95 (–1.71, –0.19)	0.01
Whole vegetables				
DASH subscore	1.12 (0.25, 1.99)	0.01	0.32 (–0.26, 0.91)	0.31
Servings per day	0.24 (–0.12, 0.59)	0.21	0.11 (–0.13, 0.35)	0.37
Whole fruits				
DASH subscore	0.19 (–0.62, 1.00)	0.63	0.08 (–0.46, 0.63)	0.69
Servings per day	–0.28 (–1.14, 0.59)	0.61	–0.52 (–1.10, 0.06)	0.09
Low-fat dairy				
DASH subscore	–0.02 (–0.75, 0.71)	0.98	0.00 (–0.49, 0.49)	0.99
Servings per day	0.48 (–0.27, 1.24)	0.19	0.26 (–0.24, 0.77)	0.30
Red and processed meat				
DASH subscore	–0.41 (–1.31, 0.48)	0.36	–0.35 (–0.96, 0.25)	0.27
Servings per day	–0.06 (–1.94, 1.82)	0.92	0.36 (–0.89, 1.62)	0.60
Nuts and legumes				
DASH subscore	–0.80 (–1.57, –0.03)	0.03	–0.14 (–0.66, 0.38)	0.52
Servings per day	0.55 (–1.00, 2.11)	0.67	0.84 (–0.20, 1.88)	0.16
Sugar-sweetened beverages				
DASH subscore	–0.73 (–1.48, 0.02)	0.06	–0.50 (–1.01, 0.00)	0.06
Servings per day	0.14 (–0.23, 0.50)	0.49	–0.12 (–0.36, 0.12)	0.34
Sodium				
DASH subscore	–0.43 (–1.86, 0.99)	0.52	–0.01 (–0.97, 0.95)	0.94
Per 1000 mg/day	1.27 (–0.67, 3.21)	0.18	–0.80 (–2.10, 0.50)	0.20

BMI, body mass index; BP, blood pressure; DASH, Dietary Approaches to Stop Hypertension; MetS, metabolic syndrome.

∗ Data are presented as β coefficient (95% confidence interval), in mm Hg, associated with each additional DASH score point or daily intake (continuous variables). Models were adjusted for sex (male, female), age (years), annual household income (< $10,000; $10,000-$24,999; $25,000-$49,999; $50,000-$74,999; $75,000-$99,999; $100,000-$149,999; $150,000-$199,999; > $200,000), BMI (kg/m^2^), smoking status (never/ever/current), alcohol consumption (grams per day), energy intake (kcal per day), physical activity level (low/moderate/high), self-reported history of high blood cholesterol (yes/no), and BP-lowering medication classes (n).

## Discussion

In this cross-sectional study nested in the CARTaGENE Québec population-based cohort, we observed no evidence of complementarity between diet quality and intensity of medication in the management of MetS-associated high BP. In fact, among younger adults (ie, men aged < 50 years and women < 60 years), as well as among individuals at lower risk of CVD per the Framingham risk score, medication was likely used as a substitute for a healthy diet, given the inverse association between diet quality and intensity of medication. The only subgroup among whom diet and medication were apparently used as complements (diet quality tended to be positively associated with medication intensity) was composed of men aged > 50 years and women aged > 60 years, who are both at higher risk of CVDs because of their age. As such, our study highlights the need for improved complementarity between diet quality and pharmacologic management of high BP in MetS in Québec.

Since the early 2000s in the Province of Québec and in Canada, the prevalence of high BP and CVDs among adults has increased by > 30%, although rates of adequate control of hypertension have declined.^11^^,^^30^ Still, approximately 80% of adults with high BP are treated according to national and provincial surveys.^11^^,^^30^ Our study sample reflects these statistics, as 75% of included participants were using at least 1 BP-lowering medication, and mean systolic and diastolic BP of individuals using medication were in the normal range. As such, although we observed no evidence of complementarity between quality of diet and intensity of medication in the management of MetS-associated high BP, our results suggest that antihypertensive medication use can effectively compensate for suboptimal dietary habits with regard to control of BP. Still, such approach raises concerns. Indeed, the use of antihypertensive medication solely addresses the clinical manifestations of MetS-associated high BP. In addition, overall diet quality at the sample level was suboptimal. Maintaining such dietary habits increases risk of CVD through long-term deterioration of cardiometabolic health, whereas it is well documented that increasing diet quality has beneficial effects on systemic metabolism and CVD risk^31^, ^32^, ^33^ regardless of the presence of concomitant weight loss.^34^^,^^35^ Second, the potential compensation of the negative effects of low diet quality on BP with medication is likely to fuel the cycle in which higher intensity of medication increases the risk of medication overuse and nonadherence, both through potential adverse effects as well as polypharmacy burden.^36^ Further analyses assessing—on the one hand—the relationship between diet quality and adherence to antihypertensive medication, and—on the other hand—the joint influence of diet quality and intensity of antihypertensive medication on incidence of CVD are needed to understand comprehensively the long-term consequences of the divergence between diet quality and use of medication in MetS-associated high BP.

The differential associations between adherence to the DASH diet and antihypertensive medication intensity we observed related to the participants’ age as well as to their 10-year risk of CVD (ie, Framingham risk score) is concordant with previously reported health literacy-related considerations in prevention of CVD.^37^, ^38^, ^39^ Overall, our results suggest that individuals who may perceive their CVD risk as being lower because of their age or their overall cardiometabolic profile are more inclined to use medication as their primary resort to manage high BP, without concomitant consideration of diet quality.^40^ Previous studies reported that some individuals found easier to take medication instead of changing their dietary habits^37^ and that preventive medication is often perceived as more effective in reducing risk of CVD than changes in lifestyle habits.^38^, ^39^, ^40^ In addition, we cannot exclude that health professionals may also have a biased perception of the long-term risk of CVD of these individuals or limited knowledge on the effects of diet on BP, which could favour a greater emphasis on medication rather than diet.^41^^,^^42^ However, information on whether participants received counselling with regard to control of BP when they were diagnosed, and, if so, what type of counselling they received was not available. Thus, whether the inverse associations we observed between diet quality and intensity of medication among younger individuals and among those at lower risk of CVD reflect that medication use acts as a barrier to healthy eating or that medication was used to compensate to individual, social, or systemic barriers to healthy eating could not be assessed in our study but remain plausible hypotheses underlying these observations. In any case, these findings highlight the need for improved access to primary care and health professionals in Québec, as well as for improved multidisciplinary frameworks to optimize prevention of CVD among at-risk individuals.

### Limitations and strengths

This study needs to be interpreted in the context of limitations and strengths. The main limitation of this work is related to the 2- to 3-year gap between BP and medication assessment (2009-2010) and the completion of the FFQ (2012). We cannot exclude that medication or diet changed during this period. However, given the direction of the associations between the DASH score and DASH subcomponents (ie, whole grains, SSB) with systolic and diastolic BP, it remains unlikely that diet significantly changed between the medication history and the FFQ completion. Second, the cross-sectional design of the study limited our ability to take into account temporal dynamics between diet and initiation of medication. Third, diet was assessed using a FFQ, which inevitably includes measurement errors because of its memory-based nature, even though it has been validated.^43^ Finally, even though BP was measured 3 times during a single visit, BP data remain exposed to random variations caused by the white-coat effect.

With regard to strengths, the sample size provided sufficient power to our main analyses. Also, our main finding on the lack of complementarity between diet quality and intensity of medication relies on analyses leveraging 3 different methods to grade BP-lowering medication. Finally, the previous demonstrations of the high agreement between self-reported medication and disease-related information provides high validity to our findings.^16^^,^^24^

## Conclusions

In this cohort of adults with MetS-associated high BP, there was an overall lack of complementarity between quality of diet and intensity of antihypertensive medication, especially among younger individuals and those with lower risk for CVD, for whom diet quality was inversely associated with intensity of medication. Our study highlights the need for improved interdisciplinary care to optimize nutritional and pharmacologic management of MetS-associated high BP in the Province of Québec.
