# Supplementary material for: Relationship Between Diet Quality and Antihypertensive Medication Intensity Among Adults With Metabolic Syndrome-Associated High Blood Pressure
Source: CJC Open. 2023 Sep 28;6(1):30–9. doi: 10.1016/j.cjco.2023.09.016 (PMC10837706; doi:10.1016/j.cjco.2023.09.016)
Supplement: Supplemental Tables and Figure [file mmc1.docx]

**Supplemental material**

**Relationship between diet quality and blood pressure-lowering medication intensity among adults with metabolic syndrome-associated high blood pressure.**

Lise Leblay, Amélie Bélanger, Clémence Desjardins, Mathieu Filiatrault, Jean-Sébastien Paquette, Jean-Philippe Drouin-Chartier

**Corresponding author**:

Jean-Philippe Drouin-Chartier

[jean-philippe.drouin-chartier@pha.ulaval.ca](mailto:jean-philippe.drouin-chartier@pha.ulaval.ca)

**Supplemental Table S1: Characteristics of the study participants according to the number of antihypertensive pills (n=915).***^1^*

| **Characteristics** | **Number of antihypertensive pills used simultaneously** | | | |
| --- | --- | --- | --- | --- |
|  | **0** | **1** | **2** | **≥3***^2^* |
| Participants, n (%) | 238 (26.0) | 472 (51.6) | 163 (17.8) | 42 (4.6) |
| Age, years | 56.7 ± 7.4 | 57.8 ± 7.3 | 58.9 ± 7.1 | 58.9 ± 6.4 |
| Sex/gender, n (%) |  |  |  |  |
| Male/men | 123 (51.7) | 231 (48.9) | 74 (45.4) | 24 (57.1) |
| Female/women | 115 (48.3) | 241 (51.1) | 89 (54.6) | 18 (42.9) |
| Annual household income, n (%) |  |  |  |  |
| Less than $49,999 | 86 (36.1) | 154 (32.6) | 72 (44.2) | 18 (42.9) |
| $50,000 - $99,999 | 88 (37.0) | 226 (47.9) | 69 (42.3) | 16 (38.1) |
| More than $100,000 | 64 (26.9) | 92 (19.5) | 22 (13.5) | 8 (19.0) |
| Self-reported history of high blood cholesterol, n (%) | 85 (35.7) | 192 (40.7) | 71 (43.6) | 13 (31.0) |
| Smoking status, n (%) |  |  |  |  |
| Never | 86 (36.1) | 207 (43.9) | 74 (45.4) | 11 (26.2) |
| Past | 115 (48.3) | 209 (44.3) | 70 (42.9) | 25 (59.5) |
| Current | 37 (15.6) | 56 (11.9) | 19 (11.7) | 6 (14.3) |
| Alcohol consumption, grams/day | 12.2 ± 18.8 | 11.5 ± 19.0 | 10.3 ± 14.6 | 12.4 ± 19.1 |
| Energy intake, kcal/day | 1,966 ± 701 | 1,942 ± 724 | 1,867 ± 637 | 1,885 ± 763 |
| Physical activity level, n (%) |  |  |  |  |
| Low | 40 (16.8) | 84 (17.8) | 34 (20.9) | 10 (23.8) |
| Moderate | 111 (46.6) | 222 (47.0) | 73 (44.8) | 12 (28.6) |
| High | 87 (36.6) | 166 (35.2) | 56 (34.4) | 20 (47.6) |
| Waist circumference, cm | 101 ± 14 | 102 ± 13 | 103 ± 12 | 108 ± 15 |
| Body mass index, kg/m² | 30.0 ± 5.4 | 30.3 ± 5.3 | 31.2 ± 5.9 | 33.8 ± 8.0 |
| Framingham risk score |  |  |  |  |
| Low (<10%) | 53 (22.3) | 125 (26.5) | 50 (30.7) | 8 (19.1) |
| Moderate (10-19%) | 97 (40.8) | 195 (41.3) | 55 (33.7) | 27 (64.3) |
| High (≥20%) | 88 (37.0) | 152 (32.2) | 58 (35.6) | 7 (16.7) |
| BP-lowering medication class, n (%) |  |  |  |  |
| Alpha adrenergic receptor^3^ | 0 (0) | 5 (1.1) | 3 (1.8) | 5 (11.9) |
| Angiotensin converting enzyme inhibitors | 0 (0) | 71 (15.0) | 38 (23.3) | 16 (38.1) |
| Angiotensin II receptor blockers | 0 (0) | 153 (32.4) | 53 (32.5) | 21 (50.0) |
| Beta-adrenergic receptor blockers | 0 (0) | 54 (11.4) | 66 (40.5) | 24 (57.1) |
| Calcium channel blockers | 0 (0) | 53 (11.2) | 66 (40.5) | 38 (90.5) |
| Diuretic | 0 (0) | 49 (10.4) | 66 (40.5) | 26 (61.9) |
| Number of BP-lowering medication classes, n (%) |  |  |  |  |
| 1 | 0 (0) | 385 (81.6) | 0 (0) | 0 (0) |
| 2 | 0 (0) | 87 (18.4) | 129 (79.1) | 1 (2.4) |
| 3 or more | 0 (0) | 0 (0) | 34 (20.9) | 41 (97.6) |
| Therapeutic intensity score*^4^* | 0 | 0.61 ± 0.45 | 0.58 ± 0.37 | 0.58 ± 0.40 |

*^1^* Continuous variables are presented as mean ± SD. Categorical variables are presented as count (percent).

*^2^* 3 classes, n=59; 4 classes, n=12; 5 classes, n=3; 6 classes, n=1.

*^3^* A total of 9/13 individuals using alpha adrenergic receptors were women.

^4^ Data on the Therapeutic Intensity Score were available for n=594/915 participants (no medication: n=238; 1 pill: n=249; 2 pills: n=86; ≥3 pills: n=21).

**Supplemental Table S2: Differences in diet quality and dietary intakes associated with each additional BP-lowering pill used concomitantly.***^1^*

| **Dietary components** | **β coefficient (95% CI)** | ***P* value** |
| --- | --- | --- |
| DASH total score | -0.05 (-0.40, 0.29) | 0.76 |
| Whole grains |  |  |
| DASH sub-score | -0.02 (-0.12, 0.08) | 0.67 |
| Servings/day | -0.02 (-0.09, 0.05) | 0.83 |
| Whole vegetables |  |  |
| DASH sub-score | -0.04 (-0.13, 0.06) | 0.46 |
| Servings/day | -0.01 (-0.25, 0.23) | 0.56 |
| Whole fruits |  |  |
| DASH sub-score | -0.06 (-0.16, 0.04) | 0.25 |
| Servings/day | -0.04 (-0.14, 0.06) | 0.22 |
| Low-fat dairy products |  |  |
| DASH sub-score | 0.10 (0.00, 0.21) | 0.06 |
| Servings/day | 0.01 (-0.09, 0.11) | 0.18 |
| Red and processed meats |  |  |
| DASH sub-score | 0.01 (-0.08, 0.10) | 0.76 |
| Servings/day | 0.02 (-0.02, 0.07) | 0.48 |
| Nuts and legumes |  |  |
| DASH sub-score | -0.05 (-0.15, 0.06) | 0.40 |
| Servings/day | -0.03 (-0.08, 0.02) | 0.40 |
| Sugar-sweetened beverages |  |  |
| DASH sub-score | -0.01 (-0.11, 0.10) | 0.85 |
| Servings/day | 0.03 (-0.21, 0.27) | 0.79 |
| Sodium |  |  |
| DASH sub-score | 0.00 (-0.06, 0.06) | 0.92 |
| mg/day | 39.3 (-8.0, 86.6) | 0.27 |

*^1^* Data are presented as β coefficient (95% confidence interval), in DASH points or daily intakes, associated with each additional BP-lowering medication pill (continuous variable). Models were adjusted for gender (men, women), age (years), annual household income (<$10,000; $10,000-24,999; $25,000-49,999; $50,000-74,999; $75,000-99,999; $100,000-149,999; $150,000-199,999; >$200,000), BMI (kg/m^2^), smoking status (never, ever, current), alcohol consumption (grams/day), energy intake (kcal/day), physical activity level (low, moderate, high) and self-reported history of high blood cholesterol (yes, no).

**Supplemental Table S3: Differences in diet quality and dietary intakes associated with the therapeutic intensity score.***^1^*

| **Dietary components** | **β coefficient (95% CI)** | ***P* value** |
| --- | --- | --- |
| DASH total score | -0.28 (-1.08, 0.53) | 0.50 |
| Whole grains |  |  |
| DASH sub-score | 0.01 (-0.24, 0.25) | 0.98 |
| Servings/day | 0.05 (-0.12, 0.22) | 0.86 |
| Whole vegetables |  |  |
| DASH sub-score | -0.23 (-0.46, 0.01) | 0.05 |
| Servings/day | -0.49 (-1.10, 0.12) | 0.08 |
| Whole fruits |  |  |
| DASH sub-score | 0.03 (-0.22, 0.28) | 0.88 |
| Servings/day | -0.08 (-0.15, 0.31) | 0.91 |
| Low-fat dairy products |  |  |
| DASH sub-score | 0.05 (-0.20, 0.31) | 0.63 |
| Servings/day | -0.12 (-0.36, 0.12) | 0.88 |
| Red and processed meats |  |  |
| DASH sub-score | 0.06 (-0.17, 0.29) | 0.64 |
| Servings/day | 0.00 (-0.12, 0.11) | 0.88 |
| Nuts and legumes |  |  |
| DASH sub-score | -0.17 (-0.42, 0.08) | 0.17 |
| Servings/day | -0.03 (-0.17, 0.11) | 0.19 |
| Sugar-sweetened beverages |  |  |
| DASH sub-score | -0.01 (-0.27, 0.24) | 0.91 |
| Servings/day | -0.24 (-0.84, 0.36) | 0.99 |
| Sodium |  |  |
| DASH sub-score | -0.01 (-0.16, 0.14) | 0.61 |
| mg/day | 85.8 (-33.4, 205.0) | 0.61 |

*^1^* The analyses included 594 participants. Data are presented as β coefficient (95% confidence interval), in DASH points or daily intakes, associated with the therapeutic intensity score (continuous variable). Models were adjusted for gender (men, women), age (years), annual household income (<$10,000; $10,000-24,999; $25,000-49,999; $50,000-74,999; $75,000-99,999; $100,000-149,999; $150,000-199,999; >$200,000), BMI (kg/m^2^), smoking status (never, ever, current), alcohol consumption (grams/day), energy intake (kcal/day), physical activity level (low, moderate, high) and self-reported history of high blood cholesterol (yes, no).

**Supplemental Table S4: Differences in dietary components of the DASH score according to BP-lowering medication use.***^1^*

| **Dietary components** | **Individuals not using BP-lowering medication (n=238)** | **Individuals using BP-lowering medication (n=677)** | ***P* value** |
| --- | --- | --- | --- |
| DASH score total | 23.5 (22.8, 24.1) | 23.4 (22.9, 23.9) | 0.88 |
| Whole grains |  |  |  |
| DASH sub-score | 2.98 (2.78, 3.18) | 2.94 (2.78, 3.10) | 0.64 |
| Servings/day | 0.77 (0.63, 0.91) | 0.77 (0.66, 0.88) | 0.79 |
| Whole vegetables |  |  |  |
| DASH sub-score | 3.02 (2.83, 3.21) | 2.87 (2.72, 3.02) | 0.10 |
| Servings/day | 4.87 (4.39, 5.34) | 4.33 (3.96, 4.70) | 0.04 |
| Whole fruits |  |  |  |
| DASH sub-score | 2.89 (2.69, 3.10) | 2.83 (2.68, 2.99) | 0.52 |
| Servings/day | 1.52 (1.33, 1.71) | 1.47 (1.32, 1.62) | 0.39 |
| Low-fat dairy products |  |  |  |
| DASH sub-score | 2.69 (2.48, 2.90) | 2.94 (2.78, 3.11) | 0.01 |
| Servings/day | 1.03 (0.83, 1.23) | 1.13 (0.97, 1.29) | 0.03 |
| Red and processed meat |  |  |  |
| DASH sub-score | 2.88 (2.70, 3.06) | 2.92 (2.78, 3.06) | 0.54 |
| Servings/day | 1.04 (0.95, 1.13) | 1.04 (0.97, 1.11) | 0.91 |
| Nuts and legumes |  |  |  |
| DASH sub-score | 2.99 (2.79, 3.20) | 2.89 (2.73, 3.05) | 0.34 |
| Servings/day | 0.57 (0.47, 0.67) | 0.48 (0.40, 0.56) | 0.21 |
| Sugar sweetened beverages |  |  |  |
| DASH sub-score | 2.99 (2.79, 3.20) | 2.96 (2.80, 3.12) | 0.76 |
| Servings/day | 1.96 (1.49, 2.42) | 1.84 (1.48, 2.21) | 0.66 |
| Sodium |  |  |  |
| DASH sub-score | 3.00 (2.88, 3.12) | 3.05 (2.96, 3.14) | 0.61 |
| mg/day | 2,824 (2,730, 2,917) | 2,866 (2,794, 2,939) | 0.73 |

*^1^* Data are presented as mean (95% confidence interval). Models were adjusted for gender (men, women), age (years), annual household income (<$10,000; $10,000-24,999; $25,000-49,999; $50,000-74,999; $75,000-99,999; $100,000-149,999; $150,000-199,999; >$200,000), BMI (kg/m^2^), smoking status (never, ever, current), alcohol consumption (grams/day), energy intake (kcal/day), physical activity level (low, moderate, high) and history of high blood cholesterol (yes, no).


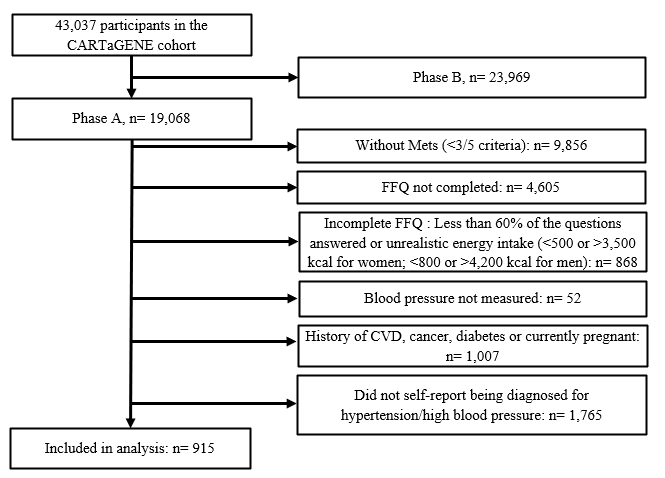


**Supplemental Figure S1: Flow-chart of participants’ selection.**
